# SELF-REPORTED AND OBJECTIVE ASSESSMENT OF HEARING HANDICAP AND COGNITIVE CHALLENGES IN AGE-RELATED HEARING LOSS

**DOI:** 10.1093/geroni/igad104.3566

**Published:** 2023-12-21

**Authors:** Shraddha Shende, Raksha Mudar

**Affiliations:** Illinois State University, Normal, Illinois, United States; University of Illinois Urbana-Champaign (UIUC), Champaign, Illinois, United States

## Abstract

Age-related hearing loss (ARHL) is a common condition in older adults, with most reporting challenges in recognizing speech-in-noise (SiN). Growing evidence also indicates cognitive alterations in this population. Concomitant examination of hearing and cognitive abilities using a combination of subjective and objective measures would be useful in characterizing the challenges faced by individuals with ARHL. We examined hearing abilities and cognitive functions in 15 individuals with bilateral mild-to-moderate untreated sensorineural ARHL (age: 70.4 ± 7.4 years, pure tone average: 31.0 ± 3.8 dB HL) and 15 age- and education-matched normal hearing controls (age: 65.7 ± 5.8 years, pure tone average: 15.4 ± 5.9 dB HL). We examined subjective ratings of hearing handicap (Hearing Handicap Inventory for Adults) and cognitive ability (Self-rating of Cognition in Everyday Activities), and objective measures of SiN (Quick Speech-In-Noise) and cognition (Montreal Cognitive Assessment [MoCA] and MoCA-memory index score [MoCA-MIS]). Our analysis revealed that individuals with ARHL self-reported a greater number of cognitive issues in everyday activities and had a higher hearing handicap score (p=.044) relative to controls. Additionally, the ARHL group performed significantly worse on SiN (p=.005) and cognitive screening (MoCA, p=.031; MoCA-MIS, p=.039). Taken together, these findings suggest that in addition to experiencing challenges with hearing abilities, individuals with mild-to-moderate ARHL also experience cognitive changes. Furthermore, these challenges are self-perceived as well as noticeable on objective testing. Our work points towards the consideration of self-report and objective assessment of cognition in geriatric hearing care, which can further inform the development of novel interventions.

